# Preservation solution Custodiol containing human alpha-1-antitrypsin improves graft recovery after prolonged cold ischemic storage in a rat model of heart transplantation

**DOI:** 10.3389/fimmu.2023.1155343

**Published:** 2023-06-22

**Authors:** Sevil Korkmaz-Icöz, Sophia Abulizi, Kunsheng Li, Brice Korkmaz, Adrian-Iustin Georgevici, Alex Ali Sayour, Sivakkanan Loganathan, Hansa Canoglu, Matthias Karck, Gábor Szabó

**Affiliations:** ^1^ Department of Cardiac Surgery, University Hospital Heidelberg, Heidelberg, Germany; ^2^ Department of Cardiac Surgery, University Hospital Halle (Saale), Halle, Germany; ^3^ INSERM UMR-1100, “Research Center for Respiratory Diseases” and University of Tours, Tours, France; ^4^ Department of Anaesthesiology, St. Josef Hospital, Ruhr-University Bochum, Bochum, Germany; ^5^ Heart and Vascular Center, Semmelweis University, Budapest, Hungary

**Keywords:** heart transplantation, ischemia/reperfusion, alpha-1-antitrypsin, cardioprotection, prolonged cold ischemic storage

## Abstract

**Introduction:**

The shortage of available donor hearts and the risk of ischemia/reperfusion injury restrict heart transplantation (HTX). Alpha-1-antitrypsin (AAT), a well-characterized inhibitor of neutrophil serine protease, is used in augmentation therapy to treat emphysema due to severe AAT deficiency. Evidence demonstrates its additional anti-inflammatory and tissue-protective effects. We hypothesized that adding human AAT in a preservation solution reduces graft dysfunction in a rat model of HTX following extended cold ischemic storage.

**Methods:**

The hearts from isogenic Lewis donor rats were explanted, stored for either 1h or 5h in cold Custodiol supplemented with either vehicle (1h ischemia, n=7 or 5h ischemia, n=7 groups) or 1 mg/ml AAT (1h ischemia+AAT, n=7 or 5h ischemia+AAT, n=9 groups) before heterotopic HTX. Left-ventricular (LV) graft function was evaluated *in vivo* 1.5h after HTX. Immunohistochemical detection of myeloperoxydase (MPO) was performed in myocardial tissue and expression of 88 gene quantified with PCR was analyzed both statistical and with machine-learning methods.

**Results:**

After HTX, LV systolic function (dP/dt_max_ 1h ischemia+AAT 4197 ± 256 vs 1h ischemia 3123 ± 110; 5h ischemia+AAT 2858 ± 154 vs 5h ischemia 1843 ± 104mmHg/s, *p*<0.05) and diastolic function (dP/dt_min_ 5h ischemia+AAT 1516 ± 68 vs 5h ischemia 1095 ± 67mmHg/s, *p*<0.05) at an intraventricular volume of 90µl were improved in the AAT groups compared with the corresponding vehicle groups. In addition, the rate pressure product (1h ischemia+AAT 53 ± 4 vs 1h ischemia 26 ± 1; 5h ischemia+AAT 37 ± 3 vs 5h ischemia 21 ± 1mmHg*beats/min at an intraventricular volume of 90µl; *p*<0.05) was increased in the AAT groups compared with the corresponding vehicle groups. Moreover, the 5h ischemia+AAT hearts exhibited a significant reduction in MPO-positive cell infiltration in comparison to the 5h ischemia group. Our computational analysis shows that ischemia+AAT network displays higher homogeneity, more positive and fewer negative gene correlations than the ischemia+placebo network.

**Discussion:**

We provided experimental evidence that AAT protects cardiac grafts from prolonged cold ischemia during HTX in rats.

## 1 Introduction

Heart transplantation (HTX) is the definitive surgical solution for patients suffering from end-stage heart failure. Although significant advancements have been made, the shortage of available donor hearts has restricted the number of HTXs performed. Currently, *ex vivo* organ storage in preservation solutions permits a safe ischemic time up to 4h regarding graft survival rates (1, 2). However, there are situations where the ischemic time must be extended unavoidably (3). Prolonged cold ischemia is thought to exacerbate ischemia/reperfusion (IR) injury. Preventing the adverse effects of myocardial IR injury, which is a main mechanism of allograft injury during the transplant procedure, from prolonged ischemia may allow expanding the donor pool by increasing the time available for transportation. The main mechanisms of IR injury-induced graft dysfunction are adenosine triphosphate (ATP) depletion of cardiomyocytes, intracellular calcium overload, the development of metabolic acidosis, cell damage by free radicals, the generation of inflammatory cytokines, and neutrophils infiltration (4). These alterations enhance cell death (5) and may lead to graft dysfunction. Therefore, it is believed that neutrophils might be involved in the early events of inflammation and participate in reperfusion injury. Taken together, prolonged ischemia times in donor hearts followed by warm blood reperfusion may result in a higher incidence of primary graft failure.

Alpha-1 antitrypsin (AAT), also known as alpha 1-proteinase inhibitor or SERPINA1, demonstrated strong inhibitory capacity against neutrophil serine proteases. It protects tissues against the action of proteolytic enzymes released from neutrophil granules, such as neutrophil elastase, cathepsin G, and proteinase 3 (6–8). This acute phase protein is mainly synthesized by liver hepatocytes, and its serum concentrations can increase up to fourfold within hours during instances of inflammatory response or infection (9). AAT is currently used clinically to treat emphysema due to AAT deficiency (10). Accumulating data suggested that, aside from its ability to inhibit proteolytic enzymes, AAT has additional highly effective anti-inflammatory (7, 11) and anti-apoptotic (12) actions, and can reduce ROS production (13). The protective effects of AAT on experimental IR-induced lung (14, 15) and renal (16) injuries have been demonstrated. Additionally, AAT has been shown to reduce inflammation and enhance survival in experimental islet-transplantation model (17). Furthermore, AAT prevented adverse post-myocardial infarction cardiac remodeling (18). Recently, we have shown that AAT protects vascular grafts of brain-dead rats from IR injury (19). However, the addition of AAT to transport solutions during cold stasis is a novel concept that we investigated in our rat model of heterotopic HTX. Utilizing registered, safe drugs or investigational products for a new purpose can be a quicker and potentially more cost-effective alternative to developing entirely new drugs.

We hypothesized that addition of human AAT to a preservation solution Custodiol, attenuates left-ventricular (LV) graft dysfunction after prolonged cold ischemia storage in a rat model of HTX.

## 2 Materials and methods

### 2.1 Animals and ethical issues

Male Lewis rats (250-350g; Janvier Labs, Saint Berthevin, France) were housed in standard temperature and light-controlled rooms (22 ± 2°C with 12-12h light-dark cycles), had access to food and water *ad libitum*. They were acclimatized for 1 week prior the experiment. The humane care of all animals was ensured in accordance with the “Principles of Laboratory Animal Care” set forth by the National Society for Medical Research, the “Guide for the Care and Use of Laboratory Animals” published by the Institute of Laboratory Animal Resources and endorsed by the National Institutes of Health (NIH Publication, 8th Edition, 2011), and the EU Directive 2010/63/EU.

### 2.2 Preparation of AAT

Before the experiment, the stock AAT (Prolastin® 1000 mg, Grifols, Frankfurt am Main, Germany) was dissolved in Custodiol to obtain a final concentration of 1 mg/mL. An equal volume of human® albumin (stock solution: 200 g/l; Grifols, Frankfurt am Main, Germany) was used as a vehicle. The concentration of AAT was selected based on previously published data (15).

### 2.3 Rat model of heterotopic HTX

#### 2.3.1 Experimental groups

The rats were divided into four groups: the donor hearts were arrested, explanted, and stored either for 1h or 5h in cold preservation solution Custodiol, supplemented with human albumin vehicle (1h ischemia, n=7 or 5h ischemia, n=7 groups), or with 1 mg/ml AAT (1h ischemia+AAT, n=7 or 5h ischemia+AAT, n=9 groups). Following preservation, the hearts were heterotopically transplanted.

#### 2.3.2 Surgical technique for HTX

The transplantations were conducted in an isogenic Lewis to Lewis rat strain model, as detailed in previous reports (20, 21). Briefly, the donors were anesthetized in a chamber using 3% isoflurane gas, maintained by inhalation through a connected tube with 1.75-2.5% in O_2_, and heparinized at a dose of 400 IU/kg. Cardiac arrest was initiated by Custodiol solution (Dr. Franz Köhler, Chemie GmbH, Bensheim, Germany). Next, the superior and inferior caval veins, as well as the pulmonary veins, were tied en masse with a 4-0 single silk suture. The heart was then explanted and immediately placed into Custodiol solution (4°C). The recipient rats were anaesthetized using the method described above and subsequently heparinized (400 IU/kg). The donor heart’s aorta and the pulmonary artery were anastomosed end-to-side to the recipient rat’s abdominal aorta and the vena cava, respectively. To minimize variability between surgical procedures, the time between explantation and reperfusion was set to either 1h or 5h depending on the experimental groups. After the anastomoses were completed, the heart was reperfused with blood *in situ* for 1h.

#### 2.3.3 Functional measurement in the graft

As described in a previous study (20), a 3F latex balloon catheter (Edwards Lifesciences Corporation, Irvine, CA, USA) was inserted through the apex into the left-ventricle and connected to a precision-calibrated syringe for fluid administration and withdrawal 1h after HTX. Furthermore, LV systolic pressure (LVSP), maximal slope of the systolic pressure increment (dP/dt_max_) and maximal slope of the diastolic pressure decrement (dP/dt_min_) at various LV volumes were measured by inserting a Millar micromanometer (Millar Instruments, Houston, TX, USA) into the left-ventricle. The LV volumes were calculated as the volume of saline administered into the balloon plus the volume of the empty balloon (0.02 ml). To obtain a complete pressure-volume curve, data were collected through incremental increases in LV volume of 0.03 ml until a volume of 0.17 ml was reached.

### 2.4 Immunohistochemical staining

Myocardial tissue samples were fixed in a 4% buffered paraformaldehyde solution and then embedded in paraffin. Five-µm thick sections were placed on adhesive slides and assessed for myeloperoxidase (MPO) immunoreactivity using a 1:200 dilution of an antibody from Abcam, Cambridge, UK. The digital images were captured using a conventional light microscope, and MPO-positive cells were counted. The evaluation was performed in a blinded manner by two examiners, from four random non-overlapping fields per myocardium per rat, and the average value was calculated.

### 2.5 Gene expression analysis

The expression of 88 genes involved in inflammation, apoptosis, and oxidative stress was profiled using RT^2^ Profiler™ PCR Array after HTX (**Online Table 1**), as they have been previously identified as key genes in these processes. As previously reported (22), total RNA was extracted from LV myocardial samples using RNe-asy Mini Kit (Qiagen, Hilden, Germany) and was reverse-transcribed into cDNA using the RT2 First Strand Kit, mixed with RT^2^ qPCR Master Mix containing SYBR Green, according to manufacturer’s instructions (Qiagen, Hilden, Germany). In this Custom Array the following non-regulated genes (genes-of interest) were used for normalization in the fold change expression data calculations: ribosomal protein lateral stalk subunit P1 (*Rplp1*), actine beta (*ACTB*), beta-2 microglobulin (*B2m*), hypoxanthine-guanine phosphoribosyl transferase-1 (*Hprt1*), and lactate dehydrogenase A (*Ldha*). Statistical significance level alpha is 0.05. Fold regulation > 2 indicates increase in gene expression, whereas < -2 shows decrease in mRNA expression.

**Table 1:** Significantly altered genes along with both corresponding p-values and fold regulation.

Phenomena	5h ischemia vs. 1h ischemia			1h ischemia + AAT vs. 1h ischemia			5h ischemia + AAT vs. 5h ischemia			5h ischemia + AAT vs. 1h ischemia + AAT		
	Symbol	*p*-Value	Regulation	Symbol	*p*-Value	Regulation	Symbol	*p*-Value	Regulation	Symbol	*p*-Value	Regulation
Apoptosis and cell death	*Aifm1*	0.014	-1.30	*Aifm1*	0.009	-1.29				*Bax*	0.008	-1.10
	*Bad*	0.003	-1.54									
	*Bcl2*	0.010	-1.13									
	*Casp3*	0.002	1.86									
	*Casp9*	0.034	-1.28									
	*Cflar*	0.044	1.81									
	*Cycs*	0.009	-1.25									
	*Fadd*	0.045	1.92									
	*Fos*	0.014	2.29									
Oxidative stress	*Ccs*	0.006	-1.31	*Nox4*	0.033	-1.65	*Cyba*	0.022	1.40	*Cyba*	0.022	1.36
	*Gpx1*	0.022	-1.18							*Txnrd2*	0.025	-1.25
	*Gpx3*	0.005	-1.37									
	*Gpx4*	0.019	-1.15									
	*Gpx7*	0.009	-1.80									
	*Gstk1*	0.003	-1.25									
	*Hspa1a*	0.004	3.70 ^B^									
	*Ncf1*	0.025	-1.28									
	*Nox4*	0.005	-1.66									
	*Prdx1*	0.003	-1.21									
	*Prdx2*	0.028	-1.14									
	*Prdx3*	0.043	-1.14									
	*Sod3*	0.039	-1.34									
	*Txnrd1*	0.002	2.33									
Inflammatory response	*Ccl2*	0.009	3.92	*Ccl20*	0.044	1.68	*Ccl11*	0.013	3.00 ^B^	*Ccl20*	0.006	-2.05
	*Icam1*	0.045	2.40	*Vegfc*	0.040	-1.47	*Vcam1*	0.041	-2.35	*CxCr4*	0.025	1.67
	*Tlr4*	0.002	-5.85 ^A^									
	*Tollip*	0.026	1.24									
	*Vegfc*	0.022	-1.48									

The significance cut-off criteria were set at p-value less than 0.05 with a fold regulation greater than 2.0 or less than 2.0. A fold regulation value above 2 denotes an increase in gene expression, while a value below -2 suggests a decrease. Refer to **Online Table 1** for gene abbreviations and **Online Table 2** for all 88 tested genes. Genes are categorized as “A” indicating that their average threshold cycle is relatively high (> 30) in either the control or test sample but reasonably low in the other sample (< 30); “B” indicating that their average threshold cycle is relatively high (> 30), meaning that their relative expression level is low in both control and test samples, and the p-value for the fold-change is either unavailable or relatively high (p > 0.05). AAT indicates alpha-1-antitrypsin.

### 2.6 Myocardial gene expression explored with non-linear machine learning methods

We used Boruta (23), a machine-learning method based on stochastic decision trees to identify and estimate the association strength between gene expressions. By iterating Boruta over all genes, we obtained multivariate CrossBoruta interaction networks, using a stress majorization algorithm to compute the geometry of the network (24). Finally, to better interpretability, the gene-gene Spearman’s correlations were color-coded. The main advantage of this method is its integrative exploration of both linear and non-linear gene-gene associations. Uniform Manifold Approximation and Projection for Dimension Reduction UMAP (25) is the second machine-learning exploration applied on our gene expressions. UMAP projects a higher-dimensional space into a lower number of dimensions, similar to principal component analysis. The main benefit of UMAP is the ability to visualize non-linear patterns from high-dimensional data in two dimensions. The entire machine-learning analysis was performed using the open-source R programming language version 3.6.1, and the source code is available upon request.

### 2.7 Statistical analysis

The data are expressed as mean ± standard error of the mean (SEM). Statistical analyses were conducted using GraphPad Prism 7.02 software (GraphPad Sofware, Inc., CA, USA). After applying Shapiro-Wilk test, if the variables had Gaussian distributions, then two-sample t-tests were performed for differences between groups, else the analysis was performed using the Mann-Whitney test. If the assumption of normality was violated, a nonparametric Mann-Whitney test was applied. For the evaluation of graft function following HTX, a two-factor mixed ANOVA (independent factor: AAT treatment and dependent factor: LV volume) and Tukey’s post-hoc test were carried out for multiple comparisons. In the case of PCR-Array gene expression, p-values were determined through a Student’s t-test of the replicate 2^(-Delta Ct) values for each gene. A value of *p* < 0.05 was considered significant.

## 3 Results

### 3.1 Effect of AAT on left-ventricular systolic and diastolic function after HTX

**Figure 1:**
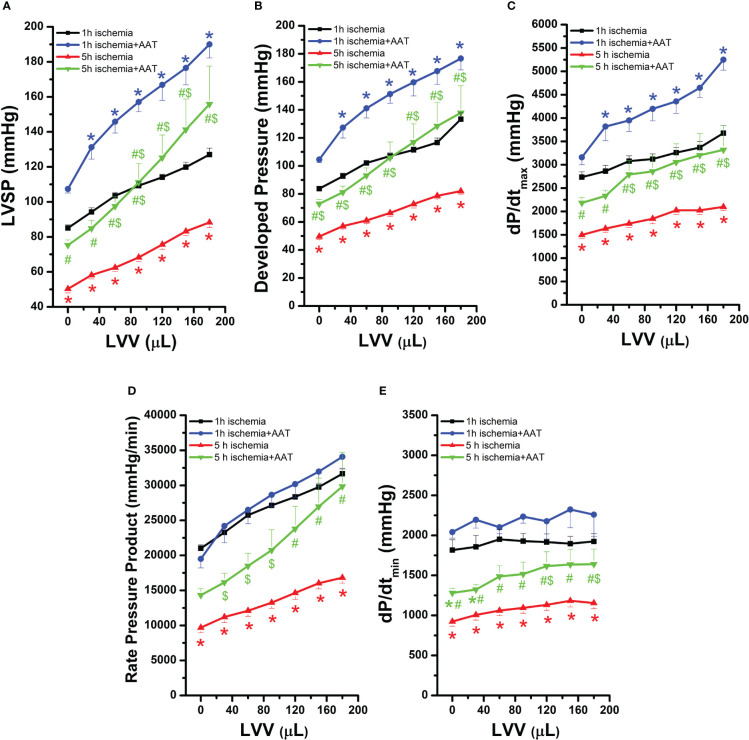
Effect of alpha-1-antitrypsin (AAT) on left-ventricular systolic and diastolic function in grafts after prolonged ischemia. **(A)** LV systolic pressure (LVSP), **(B)** developed pressure, **(C)** maximal slope of systolic pressure increment (dP/dt_max_), **(D)** rate pressure product, and **(E)** maximal slope of diastolic pressure decrement (dP/dt_min_). Values are presented as mean ± SEM. *p < 0.05 vs. 1h ischemia, ^#^ *p* < 0.05 vs. 1h ischemia+AAT, ^$^ *p* < 0.05 vs. 5h ischemia. n=7-9 rats/group.

After HTX, reduced LVSP, developed pressure, dP/dt_max_ and rate pressure product were observed in the 5h ischemia hearts compared to the 1h ischemia group, indicating decreased systolic function (**Figures 1A–D**). In both 1h ischemia and 5h ischemia groups, AAT led to improved systolic functional recovery of the grafts as compared to their corresponding controls (**Figures 1A–C**). The rate pressure product, which is used to determine the myocardial workload, was significantly increased only in the 5h ischemia+AAT hearts compared to the 5h ischemia group (**Figure 1D**). Furthermore, after HTX, reduced dP/dt_min_ in the 5h ischemia hearts compared to the 1h ischemia group was significantly improved by AAT (**Figure 1E**).

### 3.2 Effect of AAT on neutrophil infiltration after HTX

**Figure 2:**
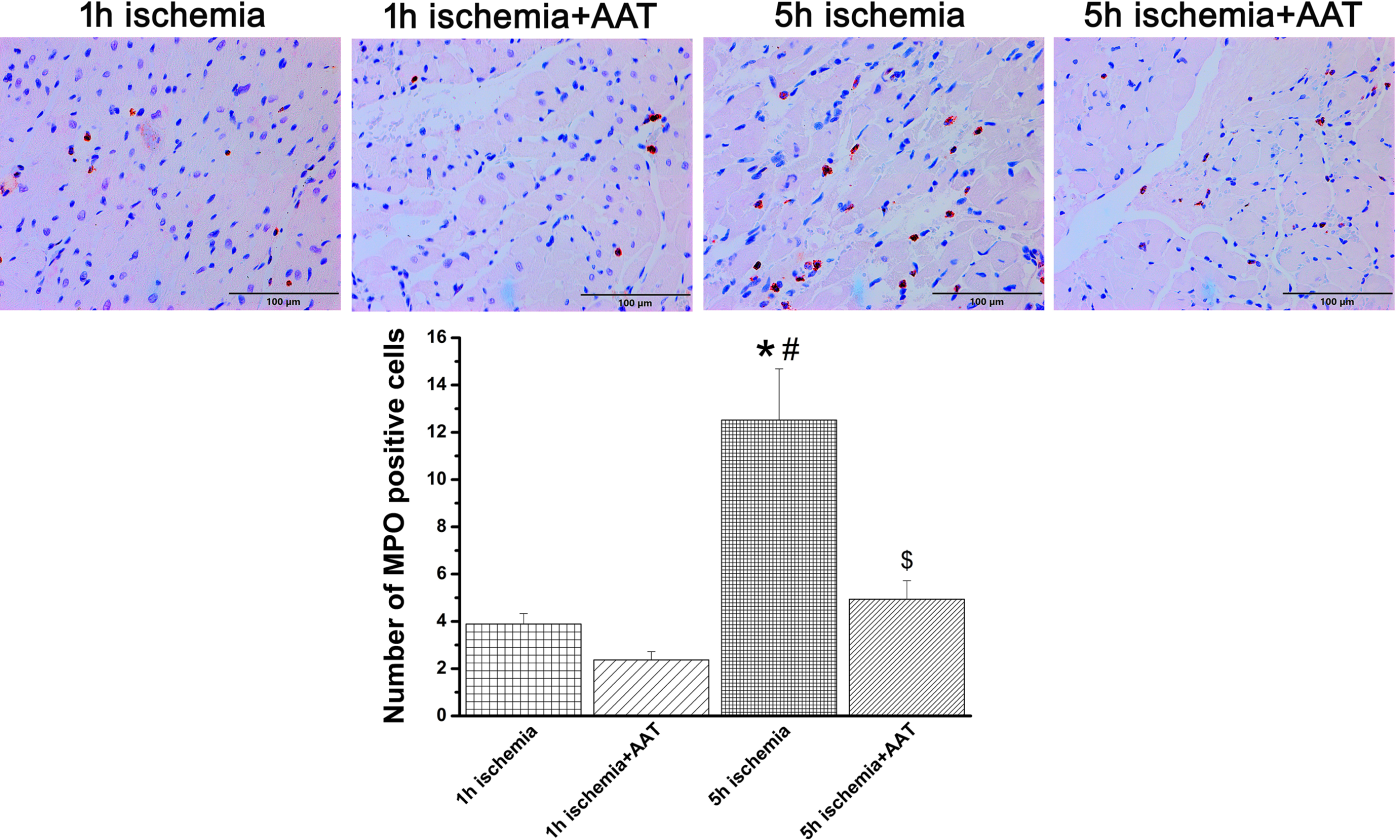
Effect of alpha-1-antitrypsin (AAT) on neutrophil infiltration in grafts after prolonged ischemia. Representative images of myeloperoxidase immunohistochemical staining (MPO, x200 magnification, scale bar: 100 µm), followed by quantification of MPO-positive cells. Values are presented as mean ± SEM. *p < 0.05 vs. 1h ischemia, ^#^ *p* < 0.05 vs. 1h ischemia+AAT, ^$^ *p* < 0.05 vs. 5h ischemia. n=6-8 rats/group.

Immunohistochemical data showed that increased myocardial neutrophil infiltration, as evidenced by quantification of MPO-positive cells, in the 5h ischemia hearts compared to the 1h ischemia group, was significantly decreased by preservation with AAT (**Figure 2**).

### 3.3 Effect of AAT on myocardial gene expression after HTX

RT^2^ PCR Profiler™ was utilized to simultaneously survey the expression levels of 88 genes associated with apoptosis, inflammatory response, and oxidative stress to investigate the effect of prolonged donor ischemic time and IR-induced cardiac changes. Among them, 5h of prolonged cold ischemia altered the expression of 6 genes compared to the 1h ischemia group (5 genes were upregulated: *ccl2*, *Fos*, *Icam1*, *Txnrd1*, *Hspa1a* and 1 gene downregulated: *Tlr4*) (**Table 1** /first column). In the 5h ischemia+AAT group, compared to 5h ischemia hearts, *Ccl11* mRNA expression was upregulated while *Vcam1* was downregulated, and these altered 6 genes were not found (**Table 1** /third column). We did not note any changes in gene expression in the 1h ischemia+AAT hearts compared to the 1h ischemia group (**Table 1** /second column).

### 3.4 Myocardial gene expression explored by machine-learning graphs

**Figure 3:**
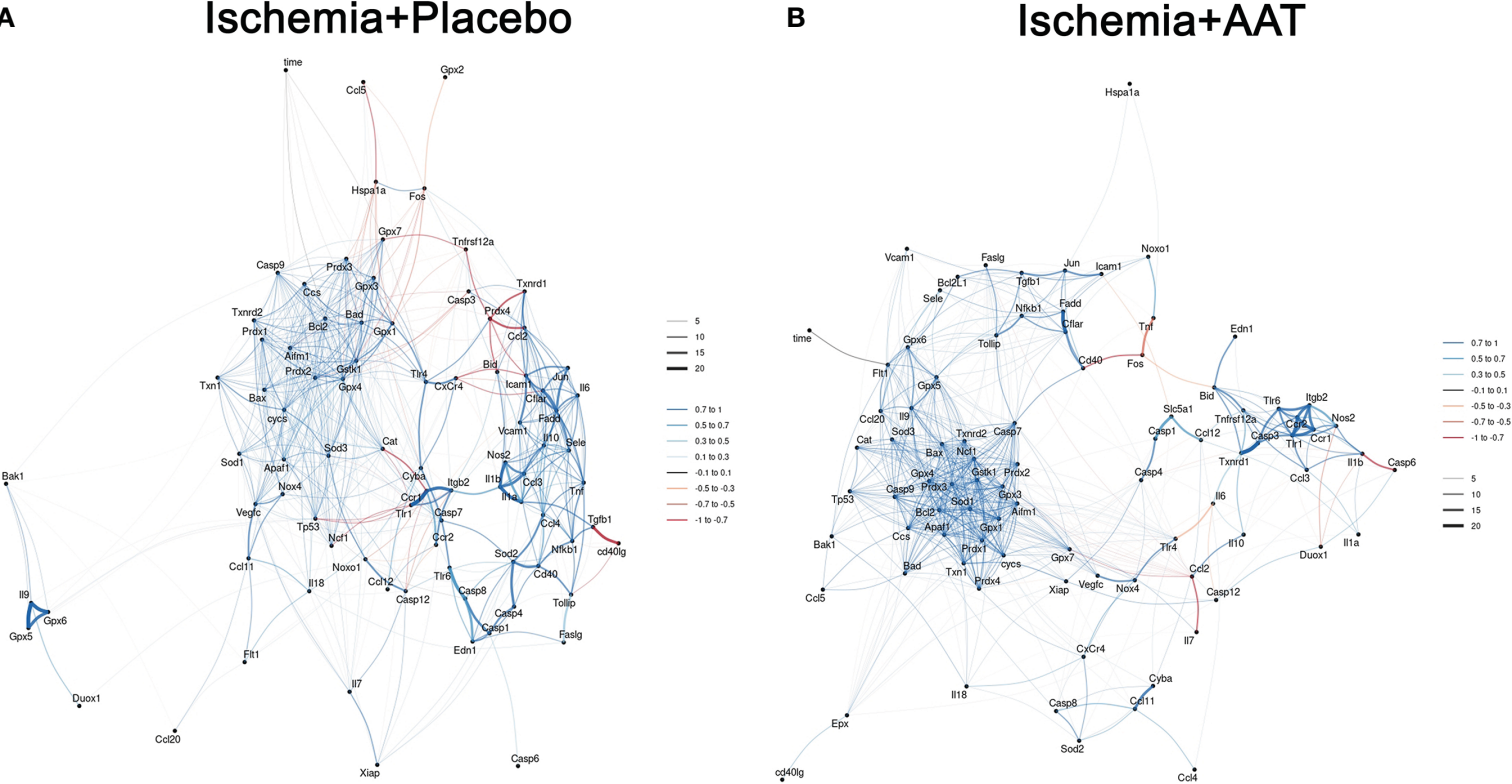
Machine learning-based Boruta networks of gene-gene interactions per study arm. Gene correlation network **(A)** Ischemia groups and **(B)** alpha-1-antitrypsin (AAT) groups. Each gene investigated is represented by a node, with the closeness between nodes and the connection width being proportional to the random-forest variable importance. Spearman’s coefficients are indicated by color, with blue or red lines representing positive or negative correlations, respectively. Connections colored in grey represent random-forest patterns that do not show a significant Spearman coefficient. Refer to Online **Table 1** for gene abbreviations. AAT indicates alpha-1-antitrypsin.

We performed a computational analysis using Crossboruta on the gene expression profiles. The network of genes that are predictive for inter-group classification (Ischemia+Placebo and Ischemia+AAT groups) is illustrated in **Figure 3**. Our results showed that the ischemia+AAT network (**Figure 3B**) shows higher homogeneity of correlations, more positive correlations (indicated by blue lines) and fewer negative correlations (indicated by red lines) than the ischemia+Placebo network (**Figure 3A**). Furthermore, *Il9*, *Gpx5*, and *Gpx6* were completely disconnected from the main gene cluster in the ischemia+Placebo network (**Figure 3A**).

**Figure 4:**
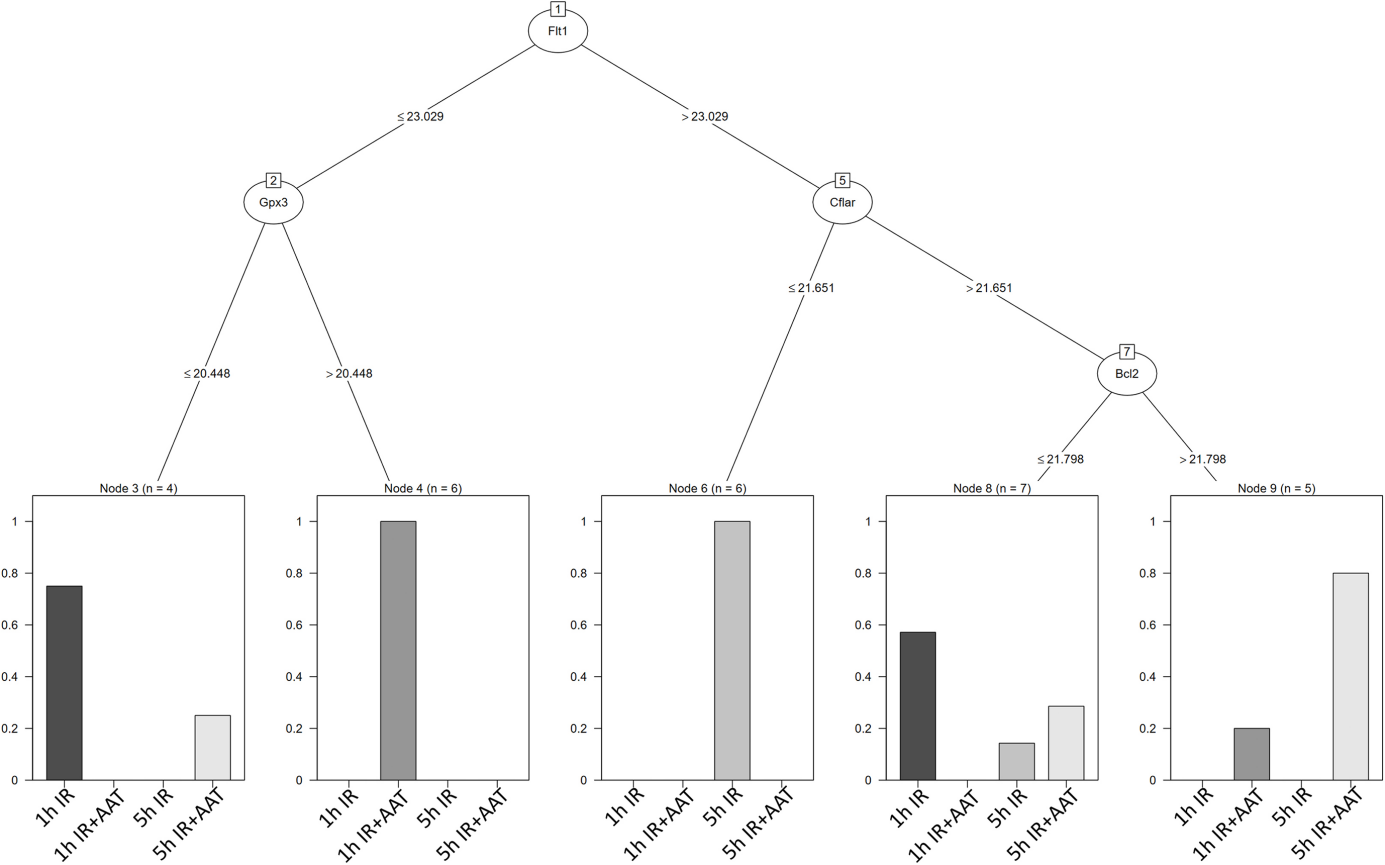
Myocardial gene expression analyzed using modern statistical and machine learning algorithms. The decision tree C5.0 display the splitting criteria employed in the inter-group classification. 1) if *Flt1* expression is ≤ 23.029 and *Gpx3* expression is ≤ 20.448, the cardiac graft belongs to either the 1h ischemia (1h IR) or 5h ischemia (5h IR) groups. If *Flt1* expression is ≤ 23.029 and *Gpx3* expression is > 20.448, the graft belongs to the 5h ischemia + AAT (5h IR+AAT) group. 2) If *Flt1* expression is > 23.029 and *Cflar* expression ≤ 21.651, the graft belongs to the 5h ischemia (5h IR) group. If *Flt1* expression is > 23.029 and *Cflar* expression > 21.651 and *Bcl2* ≤ 21.798, the graft belongs to either the 1h ischemia (1h IR) or 5h ischemia (5h IR) or 5h ischemia + AAT (5h IR+AAT) groups. If *Flt1* expression is > 23.029 and *Cflar* expression > 21.651 and *Bcl2* > 21.798, the graft belongs to either the 1h ischemia (1h IR) or 5h ischemia + AAT (5h IR+AAT) groups. Refer to **Online Table 1** for gene abbreviations. AAT indicates alpha-1-antitrypsin, IR ischemia/reperfusion.

Next (**Figure 4**), we utilized the C5.0 decision tree to display the criteria for splitting used in the inter-group classification: 1) if *Flt1* expression is ≤ 23.029 and *Gpx3* expression is ≤ 20.448, the cardiac graft belongs to either the 1h ischemia or 5h ischemia groups. If *Flt1* expression is ≤ 23.029 and *Gpx3* expression is > 20.448, the graft belongs to the 5h ischemia+AAT group. 2) if *Flt1* expression is > 23.029 and *Cflar* expression ≤ 21.651, graft belongs to the 5h ischemia group. If *Flt1* expression is > 23.029 and *Cflar* expression > 21.651 and Bcl2 ≤ 21.798, graft belongs to either the 1h ischemia or 5h ischemia or 5h ischemia + AAT groups. if *Flt1* expression is > 23.029 and *Cflar* expression > 21.651 and Bcl2 > 21.798, graft belongs to either the 1h ischemia or 5h ischemia+AAT groups.

## 4 Discussion

In this work, we hypothesized that AAT, a highly abundant human plasma neutrophil serine protease inhibitor developed to treat emphysema due to AAT deficiency, protects cardiac grafts after prolonged storage in rat model of HTX. To the best of our knowledge, this is the first study suggesting that adding of human AAT to Custodiol results in the enhanced functional recovery of grafts after cardioplegic arrest, *ex vivo* prolonged hypothermic storage, and *in vivo* blood reperfusion in rats.

Currently, brain-dead donor hearts are the major source of cardiac transplantation. To distinguish the specific effects of brain death in IR injury, in this study, we avoided additional factors that could influence our hypothesis. Efforts have been made to expand conventional limits for ischemic times in order to combat donor shortage (26). In the clinical settings, cold cardiac preservation time is limited to 4-6h, suggesting that ischemic times greater than 6h are associated with primary graft dysfunction (27). In rat cardiac allografts, even brief periods of cold ischemia (30 min, 1h, 1.5h), result in a significant increase in superoxide production, cardiomyocyte apoptosis, and inflammatory response (28), while prolonged cold ischemia (2h and 2.5h) leads to even greater damage (28). Taken together with previous studies, prolonged ischemic times cause severe damage to harvested organ in rats, which are more susceptible to ischemic insult than human hearts. Rapid recovery of graft function is a crucial factor for favorable long-term outcomes in HTX because significant hemodynamic changes occur during the initial post-transplant phase. Therefore, in this work, the cold ischemia time was fixed to 1h and graft function was assessed 1h after warm blood reperfusion.

To gain better insight into the molecular mechanism associated with IR-induced myocardial damage, we analyzed the expression of 88 genes using RT^2^ PCR Profiler™. Among them, the expression of *ccl2*, *Icam1, Fos*, *Txnrd1*, *Hspa1a* was upregulated, whereas *Tlr4* was down-regulated in the myocardium. Enhanced levels of CCl2 (monocyte chemoattractant protein-1), an inflammatory chemokine, induce the infiltration and activation of inflammatory cells. It has been shown that administration of a rationally designed CCL2 competitor reduced inflammatory monocyte recruitment, limited neointima formation, and attenuated myocardial IR injury in mice (29). Direct evidence for the regulation of ICAM-1, a master regulator of cellular response in inflammatory processes, in ischemic and reperfused canine myocardium has also been demonstrated (30). It is known that prolonged cold ischemia promotes IR injury in rat cardiac allografts (28). Next, we employed the decision tree algorithm, which is a method for classifying gene expression data. It identified that an increased *Flt1*expression (> 23.029) combined with a decrease in *Cflar* levels (≤ 21.651) was associated with prolonged ischemia-induced damage in our HTX model. Flt-1, also known as vascular endothelial growth factor receptor (VEGFR)-1, represents a promising target for attenuating cardiac IR injury (31). The expression CASP8 and FADD-like apoptosis regulator (cFlip), also known as Cflar, has been shown to be diminished in failing human and murine post-infarction hearts (32). In line with these observations, if *Flt1* expression is higher than 23.029 and *Cflar* levels lower than 21.651 in the present study, the cardiac grafts belong to 5h ischemia groups. Furthermore, the inflammatory response is a key player in IR injury. Prolonged ischemia-induced inflammation, evidenced in the present work by higher MPO-positive cells in line with neutrophil infiltration, may lead to functional alterations, demonstrated by decreased LV graft systolic and diastolic function, and reduced myocardial work in the grafts subjected to 5h of ischemia compared to 1h ischemia. Based on these considerations, novel preservation strategies are required to attenuate cellular injury during extended storage times to optimize the quality of the cardiac grafts and subsequently improve clinical outcomes.

Intravenous administration of AAT is available for the treatment of individuals with genetic AAT deficiency and chronic obstructive pulmonary disease (33). The potential of AAT therapy has been investigated for its potential in graft protection, making it an excellent pharmaceutical candidate in organ transplantation. In a preclinical pig lung transplant model, administration of human AAT before reperfusion in recipients improved early post-transplant lung function (34). Furthermore, in a murine model of lung transplantation, preservation with 1 mg/mL AAT improved primary graft function and reduced the inflammatory response after transplantation (15). These findings strongly together indicate other applications of AAT besides emphysema. We previously showed that human AAT was able to inhibit the activity of neutrophil elastase and cathepsin G from rat bone marrow cell lysates (19). In the present work, we assessed the effects of AAT on LV graft function in a rat heterotopic transplant model. Our results showed that systolic function was improved in both AAT groups, i.e. either graft submitted to 1h ischemia or 5h ischemia time, compared with the corresponding placebo groups. Furthermore, myocardial relaxation and rate pressure product were increased in the 5h ischemia+AAT hearts compared to the 5h ischemia group. In recent years, it has been recognized that AAT has significant anti-inflammatory properties. Our data showed that neutrophil infiltration into the myocardial tissues, as evidenced by MPO-positive cells, was decreased by AAT in grafts subjected to prolonged cold ischemia. In line with our observations, Gao et al. have shown that plasma MPO activity, an assessment of neutrophil activation, was significantly lower in AAT-treated animals than in controls (14). The upregulation of vascular cell adhesion molecule VCAM-1 contributes to this (35). Consistent with these findings, AAT significantly decreased *Vcam1* expression in 5h ischemia+AAT hearts compared to the 5h ischemia group. CCL11/Eotaxin, an important chemokine, is associated with recruitment of eosinophils into sites of inflammation. It has been shown that eosinophils improve cardiac function after IR-induced myocardial infarction (36). Positive effects of Ccl11 have also been reported in another study (37). We speculate that AAT’s protective effects against prolonged ischemia followed by reperfusion injury may be attributed to comparable mechanisms. The upregulation of *Ccl11* and downstream signaling by human AAT in the grafts submitted to prolonged ischemia could skew inflammatory response towards an anti-inflammatory state. It should be noted that all the altered genes in the 5h ischemia hearts compared to the 1h ischemia group (*ccl2*, *Icam1, Fos*, *Txnrd1*, *Hspa1a, and Tlr4*) were not found after AAT treatment (5h ischemia+AAT vs. 5h ischemia). Therefore, it is reasonable to suggest that these genes may have significant involvement in cardioprotection by AAT against myocardial IR injury. Although we do not have direct mechanistic evidence at the protein level, we have observed gene expression alterations related to oxidative stress in post-transplant grafts. Oxidative stress occurs when the production of reactive oxygen species, and/or reactive nitrogen species exceeds the degradation capacities of antioxidant defenses. In the present study, these alterations include the down regulation of anti-oxidant genes (*Gpx1*, *Gpx3*, *Gpx4*, *Gstk1*, *Prdx1*, *Prdx2*, *Prdx3*, *Sod3*), down-regulation of gene involved in protection against oxidative stress (*Ccs*), and upregulation of genes responsible for oxidative stress (*Txnrd1* and *Hspa1*) in the 5h ischemia hearts compared to the 1h ischemia group. However, after AAT treatment, these altered genes were not found in the 5h ischemia+AAT compared to 5h ischemia group. Furthermore, *Txnrd2*, gene responsible for oxidative stress, was down-regulated in the 5h ischemia+AAT compared to 5h ischemia group. Although bi-variate statistical tests were unconclusive in identifying differences across study arms, the multivariate machine-learning analysis showed characteristic gene-gene interaction patterns in the CrossBoruta network of each group. The ischemia+AAT network displays higher homogeneity and significantly more positive correlations than the ischemia+placebo network. It has been shown that the activation of Flt-1 plays a pivotal role in ameliorating myocardial IR injury  (38), and *Cflar* is a candidate for inhibiting apoptosis in the heart  (32). The C5.0 decision tree indicates that cardiac grafts from the ischemia+AAT (1h and 5h after ischemia) are associated with increased expressions of myocardial *Flt1*, *Cflar*, and *Bcl2*, hence supporting the protective effects of AAT. In the current study, we assume that preservation of hearts *in vitro* following prolonged cold ischemia with human AAT could be attributed, at least in part, to its ability to physically enter cardiomyocytes, vascular endothelial, and smooth muscle cells. This can regulate VEGFR-1 intracellular signaling pathways, death inhibitory proteins Cflar and Bcl2, and attenuate myocardial inflammation, subsequently leading to improved graft function. Nevertheless, the exact cellular and molecular mechanisms responsible for AAT’s effects on graft protection in our experimental model are still unknown.

In conclusion, AAT improves post-transplant grafts function after prolonged cold ischemia during HTX in rats. From the clinical point of view, due to organ shortage for HTX, the full utilization of all available suitable hearts could increase the HTX rate. In the case of an extended period of cold ischemia, a more efficient long-term preservation would be necessary. In the clinical setting, one promising approach could be the inclusion of human AAT in standard heart preservation solutions, among other options. However, the clinical relevance of our findings should be further and carefully evaluated.

## 5 Study limitations

First, we examined the effect of IR injury on LV graft function using a heterotopic abdominal rat HTX model, in which the graft was subjected to an extended ischemic time up to 5h. Since the left ventricle beats in an unloaded condition, it does not eject, thereby allowing a faster recovery after IR injury. Second, we did not assessed the structure and function of the right ventricular. Lastly, we reported the mRNA expression patterns but did not assess protein expression. Despite these limitations, our study provides promising evidence for the potential *in vitro* use of human AAT against prolonged cold ischemia occurring during HTX.

## Data availability statement

The original contributions presented in the study are included in the article/**Supplementary Material**. Further inquiries can be directed to the corresponding author.

## Ethics statement

The approval was obtained by the Ethical Committee of the Regional Council of Karlsruhe, Germany for Animal Experimentation (G264/17).

## Author contributions

Conception and research design: SK-I, BK, GS. Analysis and interpretation: SK-I, SA, BK, A-IG, SK-I. Data collection: SK-I, SA, KL. Writing the manuscript: SK-I, SA, BK, A-IG, AS, SL, HC, GS. Critical revision: SK-I, SA, BK, A-IG, AS, SL, HC, MK, GS. All the authors contributed to the article and approved the submitted version.
