# Supplementary material for: Preservation solution Custodiol containing human alpha-1-antitrypsin improves graft recovery after prolonged cold ischemic storage in a rat model of heart transplantation
Source: Front Immunol. 2023 Jun 22;14:1155343. doi: 10.3389/fimmu.2023.1155343 (PMC10323193; doi:10.3389/fimmu.2023.1155343)
Supplement: Supplementary file 1 [file Table_1.docx]

**Online Table 1. Gene Symbol and name**

| Symbol | Full name |
| --- | --- |
| Aifm1 | apoptosis-inducting factor, mitochondria associated 1 |
| Apaf1 | apoptotic peptidase activating factor 1 |
| Bad | Bcl2-associated agonist of cell death |
| Bak1 | Bcl2 antagonist/killer 1 |
| Bax | Bcl2-associated X protein, apoptosis regulator |
| Bcl2 | Bcl2, apoptosis regulator |
| Bcl2L1 | Bcl2- like 1 |
| Bid | BH3 interacting domain death agonist |
| Casp1 | caspase 1 |
| Casp12 | caspase 12 |
| Casp3 | caspase 3 |
| Casp4 | caspase 4 |
| Casp6 | caspase 6 |
| Casp7 | caspase 7 |
| Casp8 | caspase 8 |
| Casp9 | caspase 9 |
| Cat | catalase |
| Ccl11 | C-C motif chemokine ligand 11 |
| Ccl12 | Chemokine (C-C motif) ligand 12 |
| Ccl2 | C-C motif chemokine ligand 2 |
| Ccl20 | C-C motif chemokine ligand 20 |
| Ccl3 | C-C motif chemokine ligand 3 |
| Ccl4 | C-C motif chemokine ligand 4 |
| Ccl5 | C-C motif chemokine ligand 5 |
| Ccr1 | C-C motif chemokine receptor 1 |
| Ccr2 | C-C motif chemokine receptor 2 |
| Ccs | copper chaperone for superoxide dismutase |
| Cd40 | CD 40 molecule |
| cd40lg | CD 40 ligand |
| Cflar | CASP8 and FADD-like apoptosis regulator |
| CxCr4 | CXC motif chemokine receptor 4 |
| Cyba | cytochrome b-245 alpha chain |
| cycs | cytochrome c, somatic |
| Duox1 | dual oxidase 1 |
| Edn1 | endothelin 1 |
| Epx | eosinophil peroxidase |
| Fadd | Fas associated via death domain |
| Fas | Fas cell surface death receptor |
| Faslg | Fas Ligand |
| Flt1 | Fms related receptor tyrosine kinase 1 |
| Fos | Fos proto-oncogene, AP-1 transcription factor subunit |
| Gpx1 | glutathione peroxidase 1 |
| Gpx2 | glutathione peroxidase 2 |
| Gpx3 | glutathione peroxidase 3 |
| Gpx4 | glutathione peroxidase 4 |
| Gpx5 | glutathione peroxidase 5 |
| Gpx6 | glutathione peroxidase 6 |
| Gpx7 | glutathione peroxidase 7 |
| Gstk1 | glutathione S-transferase kappa 1 |
| Hspa1a | heat shock protein family A (Hsp70) member 1A |
| Icam1 | intercellular adhesion molecule 1 |
| Il10 | interleukin 10 |
| Il18 | interleukin 18 |
| Il1a | interleukin 1 alpha |
| Il1b | interleukin 1 beta |
| Il6 | interleukin 6 |
| Il7 | interleukin 7 |
| Il9 | interleukin 9 |
| Itgb2 | integrin subunit beta 2 |
| Jun | Jun proto-oncogene, AP-1 transcription factor subunit |
| Ncf1 | neutrophil cytosolic factor 1 |
| Nfkb1 | nuclear factor kappa B subunit 1 |
| Nos2 | Nitric oxide synthase 2 |
| Nox4 | NADPH oxidase 4 |
| Noxo1 | NADPH oxidase organizer 1 |
| Prdx1 | peroxiredoxin 1 |
| Prdx2 | peroxiredoxin 2 |
| Prdx3 | peroxiredoxin 3 |
| Prdx4 | peroxiredoxin 4 |
| Sele | selectin E |
| Slc5a1 | solute carrier family 5 member 1 |
| Sod1 | superoxid dismutase 1 |
| Sod2 | superoxid dismutase 2 |
| Sod3 | superoxid dismutase 3 |
| Tgfb1 | transforming growth factor, beta 1 |
| Tlr1 | toll-like receptor 1 |
| Tlr4 | toll-like receptor 4 |
| Tlr6 | toll-like receptor 6 |
| Tnf | tumor necrosis factor |
| Tnfrsf12a | TNF receptor superfamily member 12A |
| Tollip | roll interacting protein |
| Tp53 | tumor protein p53 |
| Txn1 | thioredoxin 1 |
| Txnrd1 | thioredoxin reductase 1 |
| Txnrd2 | thioredoxin reductase 2 |
| Vcam1 | vascular cell adhesion molecule 1 |
| Vegfc | vascular endothelial growth factor C |
| Xiap | X-linked inhibitor of apoptosis |
