# Supplementary material for: Preservation solution Custodiol containing human alpha-1-antitrypsin improves graft recovery after prolonged cold ischemic storage in a rat model of heart transplantation
Source: Front Immunol. 2023 Jun 22;14:1155343. doi: 10.3389/fimmu.2023.1155343 (PMC10323193; doi:10.3389/fimmu.2023.1155343)
Supplement: Supplementary file 2 [file Table_2.docx]

**Online Table 2. Gene expression data for all 88 tested genes, including p-values and fold regulation.** Statistical significance level alpha is 0.05. Fold regulation > 2 indicates increase in gene expression, whereas < -2 shows decrease in mRNA expression. Refer to **Table 1** for gene abbreviations. The genes are categorized as follows: “A” indicates that their average threshold cycle is relatively high (> 30) in either the control or test sample, but reasonably low in the other sample (< 30). These data imply that the gene's expression is relatively low in one sample and reasonably detectable in the other sample, suggesting that the actual fold-change value is at least as large as the calculated and reported fold-change result. However, if the p-value is greater than 0.05, the fold-change result may have greater variations. Therefore, it is crucial to validate the result for this gene using an adequate number of biological replicates. “B” indicates that their average threshold cycle is relatively high (> 30), meaning that their relative expression level is low in both control and test samples. The *p*-value for the fold-change is either unavailable or relatively high (*p* > 0.05). This fold-change result may also have greater variations. Hence, it is important to validate the result for this gene with sufficient biological replicates. “C” represents genes whose average threshold cycle is either undetermined or greater than the defined cut-off value in both samples. This indicates that their expression was undetected, making the fold-change result invalid and uninterpretable. AAT indicates alpha-1-antitrypsin.

| 5h ischemia vs.  1h ischemia | | | | 1h ischemia + AAT vs.  1h ischemia | | | | 5h ischemia + AAT vs.  5h ischemia | | | | 5h ischemia + AAT vs.  1h ischemia + AAT | | | |
| --- | --- | --- | --- | --- | --- | --- | --- | --- | --- | --- | --- | --- | --- | --- | --- |
| Symbol | ***p*-Value** | **Regulation** | **Comments** | **Symbol** | ***p*-Value** | **Regulation** | **Comments** | **Symbol** | ***p*-Value** | **Regulation** | **Comments** | **Symbol** | ***p*-Value** | **Regulation** | **Comments** |
| *Aifm1* | 0.014 | **-1.30** |  | *Aifm1* | 0.009 | **-1.29** |  | *Aifm1* | 0.857 | -1.03 |  | *Aifm1* | 0.847 | -1.03 |  |
| *Apaf1* | 0.092 | -1.21 |  | *Apaf1* | 0.427 | -1.09 |  | *Apaf1* | 0.729 | -1.05 |  | *Apaf1* | 0.254 | -1.17 |  |
| *Bad* | 0.003 | **-1.54** |  | *Bad* | 0.079 | -1.25 |  | *Bad* | 0.087 | 1.24 |  | *Bad* | 0.915 | 1.01 |  |
| *Bak1* | 0.386 | 1.08 |  | *Bak1* | 0.458 | 1.09 |  | *Bak1* | 0.633 | 1.04 |  | *Bak1* | 0.717 | 1.04 |  |
| *Bax* | 0.872 | -1.01 |  | *Bax* | 0.170 | 1.08 |  | *Bax* | 0.692 | -1.01 |  | *Bax* | 0.008 | **-1.10** |  |
| *Bcl2* | 0.010 | **-1.13** |  | *Bcl2* | 0.585 | -1.03 |  | *Bcl2* | 0.164 | 1.06 |  | *Bcl2* | 0.384 | -1.04 |  |
| *Bcl2L1* | 0.436 | 1. 20 |  | *Bcl2L1* | 0.330 | 1.27 |  | *Bcl2L1* | 0.948 | 1.01 |  | *Bcl2L1* | 0.820 | -1.05 |  |
| *Bid* | 0.052 | 1.72 |  | *Bid* | 0.188 | 1.41 |  | *Bid* | 0.699 | -1.11 |  | *Bid* | 0.730 | 1.10 |  |
| *Casp1* | 0.900 | 1.03 |  | *Casp1* | 0.625 | 1.12 |  | *Casp1* | 0.909 | -1.03 |  | *Casp1* | 0.628 | -1.12 |  |
| *Casp12* | 0.761 | 1.10 |  | *Casp12* | 0.416 | 1.24 |  | *Casp12* | 0.867 | -1.04 |  | *Casp12* | 0.335 | -1.18 |  |
| *Casp3* | 0.002 | **1.86** |  | *Casp3* | 0.204 | 1.25 |  | *Casp3* | 0.229 | -1.30 |  | *Casp3* | 0.539 | 1.14 |  |
| *Casp4* | 0.477 | 1.25 |  | *Casp4* | 0.825 | 1.06 |  | *Casp4* | 0.462 | -1.21 |  | *Casp4* | 0.893 | -1.03 |  |
| *Casp6* | 0.431 | -1.15 |  | *Casp6* | 0.190 | -1.23 |  | *Casp6* | 0.984 | -1.00 |  | *Casp6* | 0.721 | 1.07 |  |
| *Casp7* | 0.406 | -1.09 |  | *Casp7* | 0.646 | 1.04 |  | *Casp7* | 0.198 | 1.11 |  | *Casp7* | 0.674 | -1.03 |  |
| *Casp8* | 0.670 | 1.07 |  | *Casp8* | 0.649 | -1.07 |  | *Casp8* | 0.680 | -1.07 |  | *Casp8* | 0.659 | 1.07 |  |
| *Casp9* | 0.034 | **-1.28** |  | *Casp9* | 0.802 | 1.03 |  | *Casp9* | 0.284 | 1.09 |  | *Casp9* | 0.051 | -1.21 |  |
| *Cat* | 0.284 | -1.11 |  | *Cat* | 0.412 | -1.11 |  | *Cat* | 0.534 | 1.04 |  | *Cat* | 0.686 | 1.04 |  |
| *Ccl11* | 0.104 | -2.00 | B | *Ccl11* | 0.648 | -1.19 | B | *Ccl11* | 0.013 | **3.00** | B | *Ccl11* | 0.104 | 1.79 | B |
| *Ccl12* | 0.405 | -1.53 |  | *Ccl12* | 0.200 | -1.78 |  | *Ccl12* | 0.646 | 1.27 |  | *Ccl12* | 0.392 | 1.47 |  |
| *Ccl2* | 0.009 | **3.92** |  | *Ccl2* | 0.776 | 1.14 |  | *Ccl2* | 0.142 | -2.12 |  | *Ccl2* | 0.340 | 1.62 |  |
| *Ccl20* | 0.632 | 1.08 |  | *Ccl20* | 0.044 | **1.68** |  | *Ccl20* | 0.078 | -1.32 |  | *Ccl20* | 0.006 | **-2.05** |  |
| *Ccl3* | 0.134 | 2.04 |  | *Ccl3* | 0.379 | 1.35 |  | *Ccl3* | 0.961 | 1.02 |  | *Ccl3* | 0.262 | 1.55 |  |
| *Ccl4* | 0.148 | 1.83 |  | *Ccl4* | 0.602 | -1.15 |  | *Ccl4* | 0.360 | -1.45 |  | *Ccl4* | 0.186 | 1.45 |  |
| *Ccl5* | 0.925 | -1.01 |  | *Ccl5* | 0.355 | -1.13 |  | *Ccl5* | 0.742 | 1.04 |  | *Ccl5* | 0.180 | 1.15 |  |
| *Ccr1* | 0.193 | 1.79 |  | *Ccr1* | 0.336 | 1.53 |  | *Ccr1* | 0.573 | 1.26 |  | *Ccr1* | 0.346 | 1.47 |  |
| *Ccr2* | 0.947 | 1.03 |  | *Ccr2* | 0.467 | 1.40 |  | *Ccr2* | 0.318 | 1.58 |  | *Ccr2* | 0.750 | 1.16 |  |
| *Ccs* | 0.006 | **-1.31** |  | *Ccs* | 0.068 | -1.20 |  | *Ccs* | 0.365 | 1.10 |  | *Ccs* | 0.961 | 1.01 |  |
| *Cd40* | 0.311 | 1.29 |  | *Cd40* | 0.915 | 1.03 |  | *Cd40* | 0.414 | -1.19 |  | *Cd40* | 0.758 | 1.06 |  |
| *cd40lg* | 0.286 | 1.76 | B | *cd40lg* | 0.834 | -1.10 | B | *cd40lg* | 0.623 | -1.30 | B | *cd40lg* | 0.388 | 1.50 | B |
| *Cflar* | 0.044 | **1.81** |  | *Cflar* | 0.312 | 1.22 |  | *Cflar* | 0.065 | -1.60 |  | *Cflar* | 0.591 | -1.07 |  |
| *CxCr4* | 0.063 | -1.98 |  | *CxCr4* | 0.051 | -1.84 |  | *CxCr4* | 0.062 | 1.79 |  | *CxCr4* | 0.025 | **1.67** |  |
| *Cyba* | 0.176 | -1.21 |  | *Cyba* | 0.208 | -1.18 |  | *Cyba* | 0.022 | **1.40** |  | *Cyba* | 0.022 | **1.36** |  |
| *Cycs* | 0.009 | **-1.25** |  | *Cycs* | 0.052 | -1.19 |  | *Cycs* | 0.908 | -1.01 |  | *Cycs* | 0.590 | -1.07 |  |
| *Duox1* | 0.216 | 1.45 | B | *Duox1* | 0.773 | 1.10 | B | *Duox1* | 0.578 | -1.16 | B | *Duox1* | 0.694 | 1.13 | B |
| *Edn1* | 0.267 | 1.30 |  | *Edn1* | 0.365 | 1.24 |  | *Edn1* | 0.581 | 1.17 |  | *Edn1* | 0.494 | 1.22 |  |
| *Epx* | 0.342 | -1.35 | B | *Epx* | 0.942 | -1.02 | B | *Epx* | 0.916 | 1.03 | B | *Epx* | 0.451 | -1.27 | B |
| *Fadd* | 0.045 | **1.92** |  | *Fadd* | 0.229 | 1.34 |  | *Fadd* | 0.094 | -1.65 |  | *Fadd* | 0.507 | -1.15 |  |
| *Fas* | 0.224 | 1.58 | B | *Fas* | 0.511 | 1.33 | B | *Fas* | 0.481 | -1.33 | B | *Fas* | 0.810 | -1.12 | B |
| *Faslg* | 0.205 | 1.22 |  | *Faslg* | 0.703 | 1.06 |  | *Faslg* | 0.347 | -1.10 |  | *Faslg* | 0.621 | 1.05 |  |
| *Flt1* | 0.925 | 1.01 |  | *Flt1* | 0.125 | 1.16 |  | *Flt1* | 0.738 | 1.02 |  | *Flt1* | 0.140 | -1.13 |  |
| *Fos* | 0.014 | **2.29** |  | *Fos* | 0.920 | -1.04 |  | *Fos* | 0.520 | -1.25 |  | *Fos* | 0.175 | 1.92 |  |
| *Gpx1* | 0.022 | **-1.18** |  | *Gpx1* | 0.175 | -1.07 |  | *Gpx1* | 0.176 | 1.09 |  | *Gpx1* | 0.968 | -1.00 |  |
| *Gpx2* | 0.098 | 2.42 | B | *Gpx2* | 0.357 | 1.72 | B | *Gpx2* | 0.146 | -2.22 | B | *Gpx2* | 0.452 | -1.57 | B |
| *Gpx3* | 0.005 | **-1.37** |  | *Gpx3* | 0.065 | -1.19 |  | *Gpx3* | 0.797 | 1.04 |  | *Gpx3* | 0.532 | -1.10 |  |
| *Gpx4* | 0.019 | **-1.15** |  | *Gpx4* | 0.704 | -1.02 |  | *Gpx4* | 0.931 | -1.01 |  | *Gpx4* | 0.109 | -1.13 |  |
| *Gpx5* | 0.198 | 1.12 | C | *Gpx5* | 0.194 | 1.12 | C | *Gpx5* | 0.076 | -1.09 | C | *Gpx5* | 0.099 | -1.09 | C |
| *Gpx6* | 0.198 | 1.12 | C | *Gpx6* | 0.194 | 1.12 | C | *Gpx6* | 0.076 | -1.09 | C | *Gpx6* | 0.099 | -1.09 | C |
| *Gpx7* | 0.009 | **-1.80** |  | *Gpx7* | 0.102 | -1.39 |  | *Gpx7* | 0.422 | 1.23 |  | *Gpx7* | 0.849 | -1.05 |  |
| *Gstk1* | 0.003 | **-1.25** |  | *Gstk1* | 0.281 | -1.07 |  | *Gstk1* | 0.189 | 1.10 |  | *Gstk1* | 0.399 | -1.06 |  |
| *Icam1* | 0.045 | **2.40** |  | *Icam1* | 0.577 | 1.19 |  | *Icam1* | 0.064 | -1.99 |  | *Icam1* | 0.959 | 1.01 |  |
| *Il10* | 0.075 | 2.42 |  | *Il10* | 0.721 | 1.13 |  | *Il10* | 0.570 | -1.31 |  | *Il10* | 0.192 | 1.63 |  |
| *Il18* | 0.354 | -1.12 |  | *Il18* | 0.869 | 1.02 |  | *Il18* | 0.079 | 1.25 |  | *Il18* | 0.461 | 1.09 |  |
| *Il1a* | 0.770 | 1.19 |  | *Il1a* | 0.592 | -1.27 |  | *Il1a* | 0.727 | 1.22 |  | *Il1a* | 0.142 | 1.85 |  |
| *Il1b* | 0.204 | 2.16 |  | *Il1b* | 0.684 | 1.25 |  | *Il1b* | 0.869 | 1.09 |  | *Il1b* | 0.191 | 1.89 |  |
| *Il6* | 0.066 | 2.68 |  | *Il6* | 0.661 | 1.18 |  | *Il6* | 0.199 | -1.88 |  | *Il6* | 0.604 | 1.20 |  |
| *Il7* | 0.514 | -1.24 | B | *Il7* | 0.132 | -1.74 | B | *Il7* | 0.426 | -1.27 | B | *Il7* | 0.748 | 1.11 | B |
| *Il9* | 0.198 | 1.31 | B | *Il9* | 0.194 | 1.12 | C | *Il9* | 0.217 | -1.28 | B | *Il9* | 0.099 | -1.09 | C |
| *Itgb2* | 0.185 | 1.59 |  | *Itgb2* | 0.493 | 1.27 |  | *Itgb2* | 0.904 | 1.04 |  | *Itgb2* | 0.393 | 1.30 |  |
| *Jun* | 0.108 | 1.66 |  | *Jun* | 0.986 | 1.00 |  | *Jun* | 0.113 | -1.53 |  | *Jun* | 0.662 | 1.08 |  |
| *Ncf1* | 0.025 | **-1.28** |  | *Ncf1* | 0.530 | -1.09 |  | *Ncf1* | 0.871 | -1.02 |  | *Ncf1* | 0.203 | -1.20 |  |
| *Nfkb1* | 0.177 | 1.35 |  | *Nfkb1* | 0.811 | 1.04 |  | *Nfkb1* | 0.370 | -1.20 |  | *Nfkb1* | 0.587 | 1.08 |  |
| *Nos2* | 0.060 | 2.85 |  | *Nos2* | 0.357 | 1.41 |  | *Nos2* | 0.937 | 1.05 |  | *Nos2* | 0.179 | 2.13 |  |
| *Nox4* | 0.005 | **-1.66** |  | *Nox4* | 0.033 | **-1.65** |  | *Nox4* | 0.081 | 1.34 |  | *Nox4* | 0.197 | 1.34 |  |
| *Noxo1* | 0.779 | 1.10 | B | *Noxo1* | 0.067 | 1.86 | B | *Noxo1* | 0.146 | 1.79 | B | *Noxo1* | 0.864 | 1.06 | B |
| *Prdx1* | 0.003 | **-1.21** |  | *Prdx1* | 0.123 | -1.09 |  | *Prdx1* | 0.546 | 1.04 |  | *Prdx1* | 0.313 | -1.07 |  |
| *Prdx2* | 0.028 | **-1.14** |  | *Prdx2* | 0.774 | -1.02 |  | *Prdx2* | 0.747 | -1.02 |  | *Prdx2* | 0.057 | -1.14 |  |
| *Prdx3* | 0.043 | **-1.14** |  | *Prdx3* | 0.733 | -1.02 |  | *Prdx3* | 0.782 | -1.02 |  | *Prdx3* | 0.118 | -1.13 |  |
| *Prdx4* | 0.638 | -1.15 |  | *Prdx4* | 0.852 | 1.06 |  | *Prdx4* | 0.109 | 1.23 |  | *Prdx4* | 0.939 | 1.01 |  |
| *Sele* | 0.176 | 1.97 |  | *Sele* | 0.319 | 1.49 |  | *Sele* | 0.108 | -1.97 |  | *Sele* | 0.181 | -1.49 |  |
| *Slc5a1* | 0.268 | 1.68 | B | *Slc5a1* | 0.731 | -1.13 | B | *Slc5a1* | 0.511 | -1.40 | B | *Slc5a1* | 0.463 | 1.35 | B |
| *Sod1* | 0.060 | -1.14 |  | *Sod1* | 0.164 | -1.10 |  | *Sod1* | 0.659 | -1.03 |  | *Sod1* | 0.367 | -1.07 |  |
| *Sod2* | 0.187 | 1.16 |  | *Sod2* | 0.839 | -1.02 |  | *Sod2* | 0.577 | -1.06 |  | *Sod2* | 0.217 | 1.11 |  |
| *Sod3* | 0.039 | **-1.34** |  | *Sod3* | 0.066 | -1.28 |  | *Sod3* | 0.709 | 1.06 |  | *Sod3* | 0.934 | 1.01 |  |
| *Tgfb1* | 0.484 | 1.12 |  | *Tgfb1* | 0.464 | 1.10 |  | *Tgfb1* | 0.623 | -1.08 |  | *Tgfb1* | 0.636 | -1.06 |  |
| *Tlr1* | 0.125 | 1.82 |  | *Tlr1* | 0.264 | 1.51 |  | *Tlr1* | 0.643 | 1.20 |  | *Tlr1* | 0.346 | 1.45 |  |
| *Tlr4* | 0.002 | **-5.85** | A | *Tlr4* | 0.056 | -2.23 |  | *Tlr4* | 0.061 | 2.88 | A | *Tlr4* | 0.843 | 1.10 |  |
| *Tlr6* | 0.589 | 1.10 |  | *Tlr6* | 0.594 | 1.08 |  | *Tlr6* | 0.239 | 1.29 |  | *Tlr6* | 0.152 | 1.31 |  |
| *Tnf* | 0.160 | 1.86 |  | *Tnf* | 0.662 | -1.16 |  | *Tnf* | 0.222 | -1.56 |  | *Tnf* | 0.174 | 1.38 |  |
| *Tnfrsf12a* | 0.069 | 2.55 |  | *Tnfrsf12a* | 0.200 | 1.85 |  | *Tnfrsf12a* | 0.387 | -1.44 |  | *Tnfrsf12a* | 0.921 | -1.04 |  |
| *Tollip* | 0.026 | **1.24** |  | Tollip | 0.812 | -1.02 |  | *Tollip* | 0.567 | -1.08 |  | *Tollip* | 0.277 | 1.16 |  |
| *Tp53* | 0.674 | 1.05 |  | Tp53 | 0.828 | -1.02 |  | *Tp53* | 0.396 | -1.09 |  | *Tp53* | 0.896 | -1.01 |  |
| *Txn1* | 0.106 | -1.06 |  | Txn1 | 0.959 | 1.00 |  | *Txn1* | 0.958 | 1.00 |  | *Txn1* | 0.175 | -1.06 |  |
| *Txnrd1* | 0.002 | **2.33** |  | Txnrd1 | 0.084 | 1.47 |  | *Txnrd1* | 0.241 | -1.40 |  | *Txnrd1* | 0.647 | 1.13 |  |
| *Txnrd2* | 0.067 | -1.17 |  | Txnrd2 | 0.586 | 1.05 |  | *Txnrd2* | 0.849 | -1.01 |  | *Txnrd2* | 0.025 | **-1.25** |  |
| *Vcam1* | 0.131 | 2.13 |  | Vcam1 | 0.611 | 1.20 |  | *Vcam1* | 0.041 | **-2.35** |  | *Vcam1* | 0.184 | -1.32 |  |
| *Vegfc* | 0.022 | **-1.48** |  | Vegfc | 0.040 | **-1.47** |  | *Vegfc* | 0.586 | 1.09 |  | *Vegfc* | 0.645 | 1.09 |  |
| *Xiap* | 0.763 | -1.04 |  | Xiap | 0.492 | -1.11 |  | *Xiap* | 0.481 | -1.08 |  | *Xiap* | 0.871 | -1.02 |  |
| *Hspa1a* | 0.004 | **3.70** | B | Hspa1a | 0.441 | 1.50 | B | *Hspa1a* | 0.111 | -1.73 | B | *Hspa1a* | 0.473 | 1.43 | B |
